# Supplementary figures and images for: Combining biomarker and virus phylogenetic models improves HIV-1 epidemiological source identification
Source: PLoS Comput Biol. 2022 Aug 26;18(8):e1009741. doi: 10.1371/journal.pcbi.1009741 (PMC9455879; doi:10.1371/journal.pcbi.1009741)

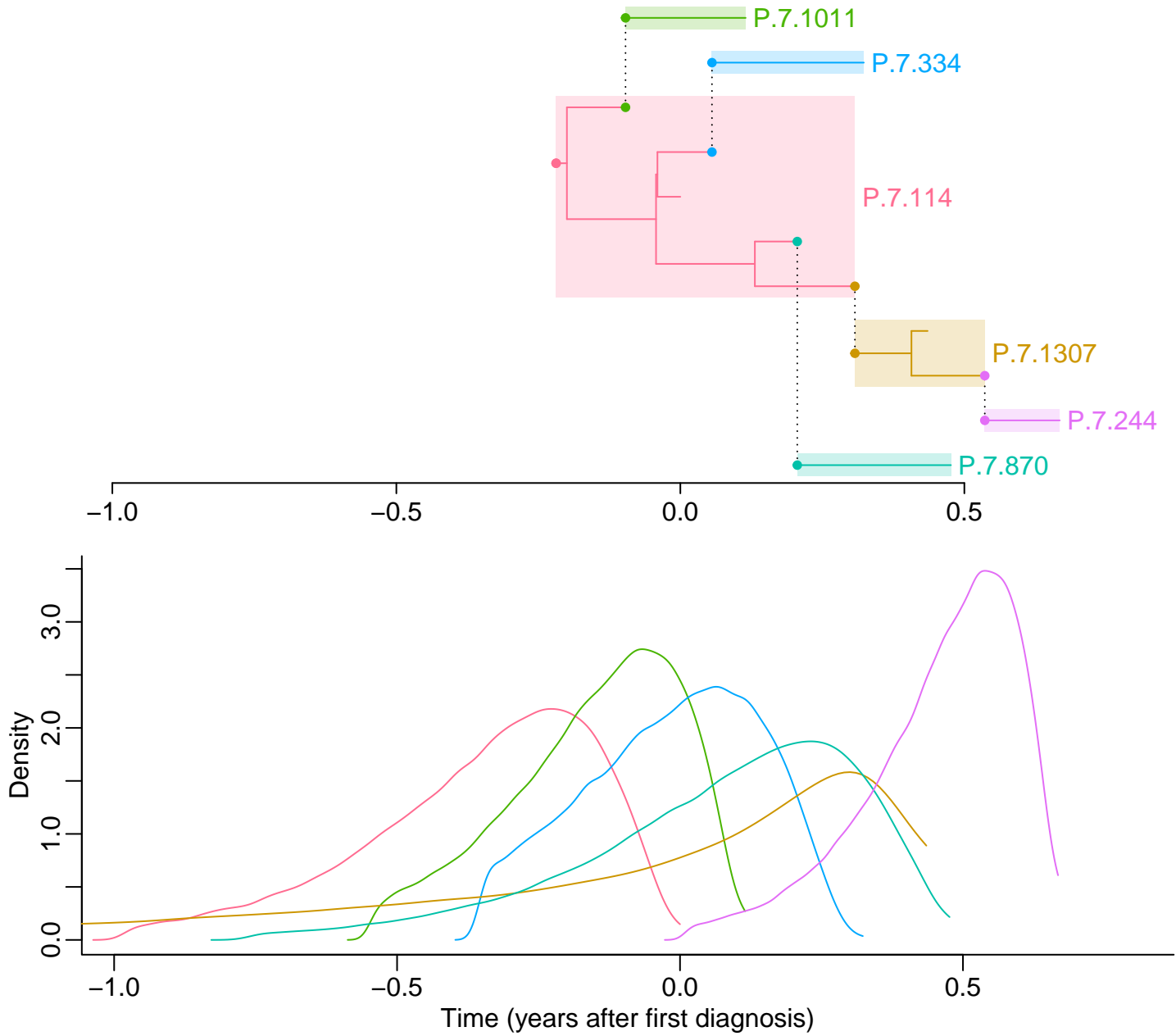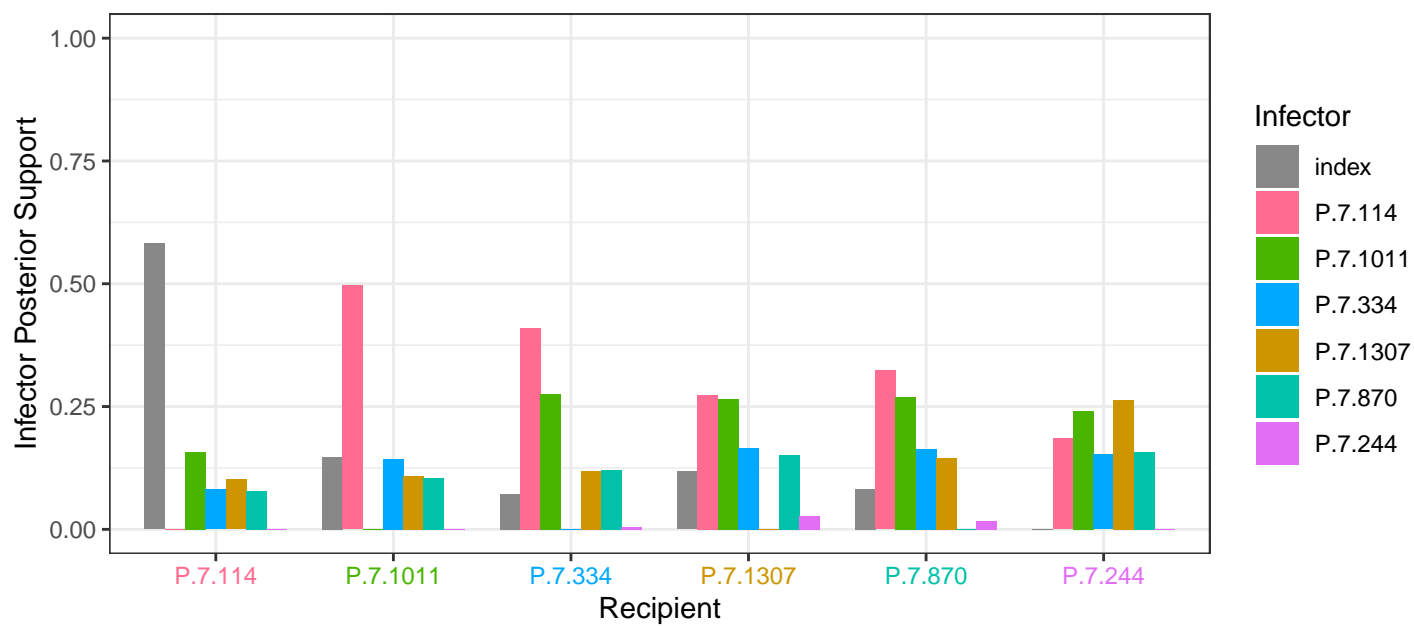

Supplement: S6 Fig — (Top) Inferred maximum parent credibility trees for each transmission cluster. (Middle) Distributions of infection times inferred from biomarker values for each individual. (Bottom) Posterior support for each individual to be the source for each individual, with the height of the colored bars represent the posterior support for the corresponding individual to be their infector. (PDF) [file pcbi.1009741.s006.pdf]

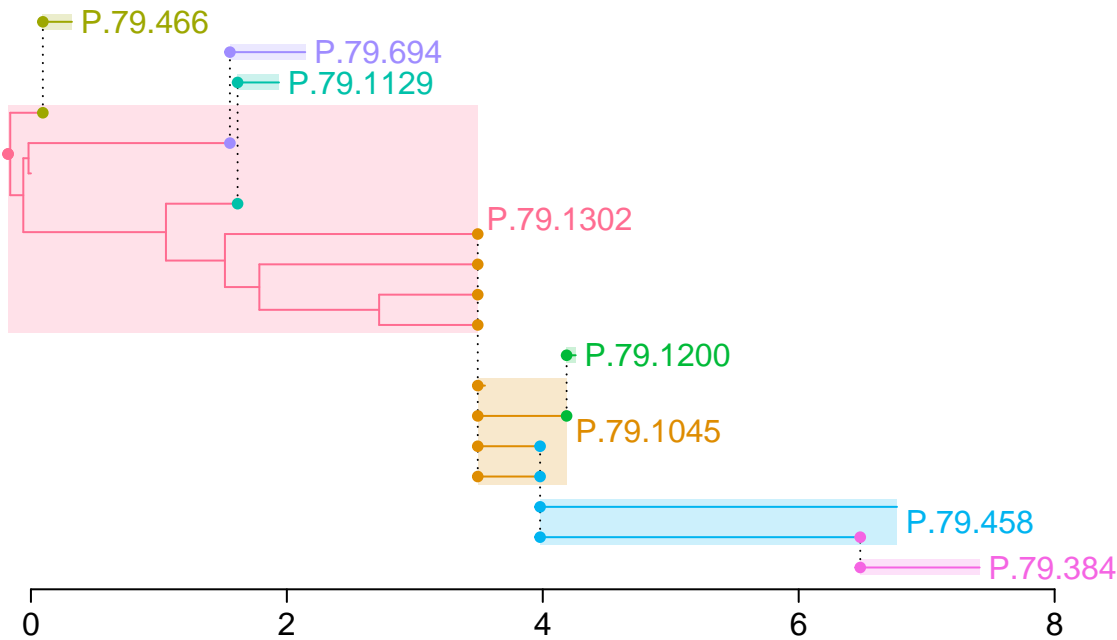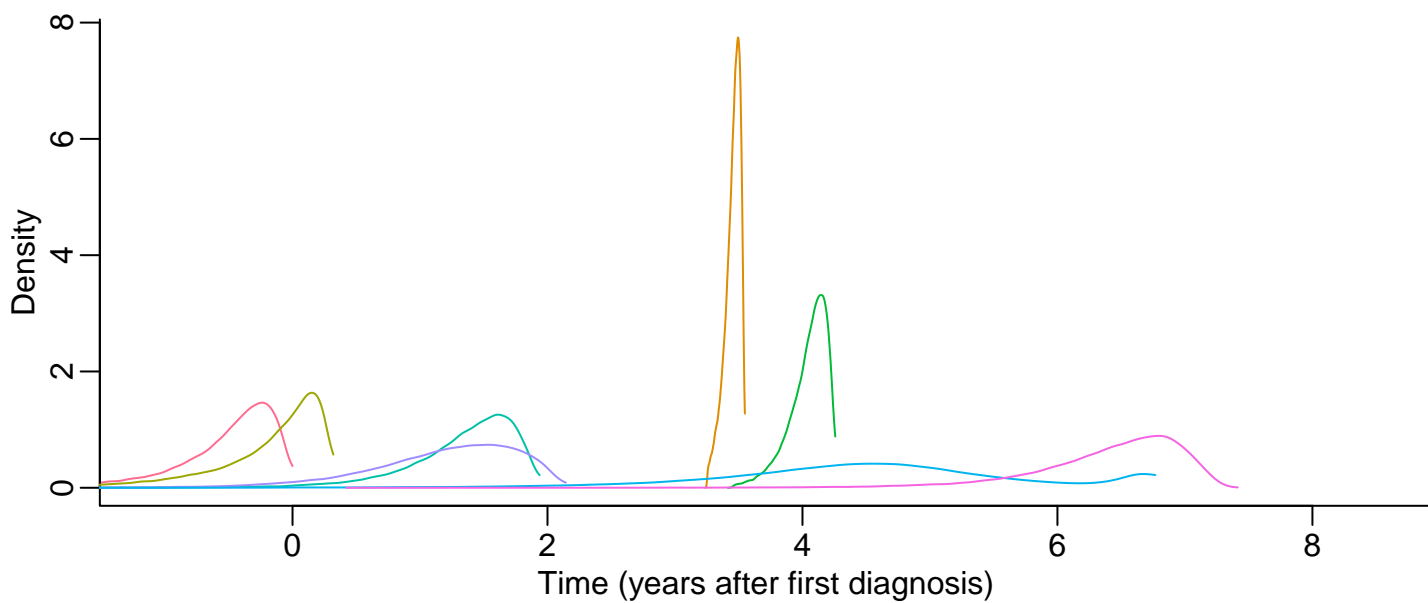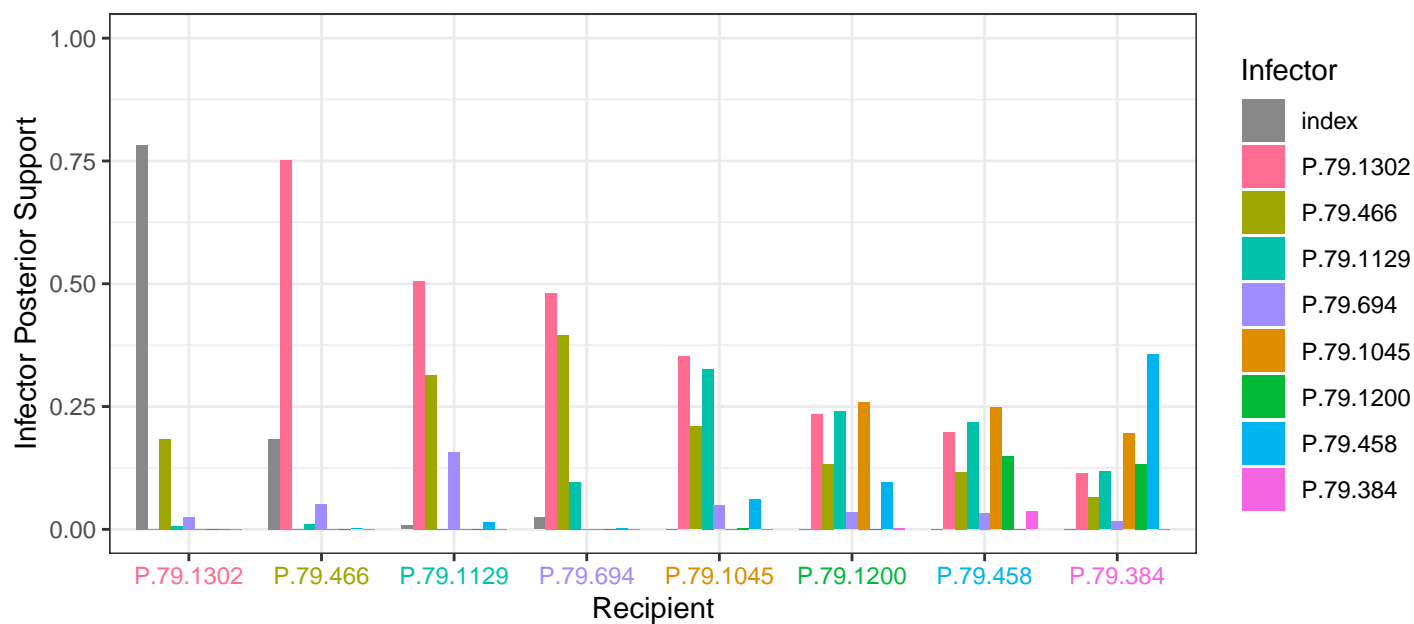

Supplement: S7 Fig — (Top) Inferred maximum parent credibility trees for each transmission cluster. (Middle) Distributions of infection times inferred from biomarker values for each individual. (Bottom) Posterior support for each individual to be the source for each individual, with the height of the colored bars represent the posterior support for the corresponding individual to be their infector. (PDF) [file pcbi.1009741.s007.pdf]
